# Extracorporeal membrane oxygenation bridge to transplant in the era of the lung composite allocation score

**DOI:** 10.1016/j.jhlto.2025.100273

**Published:** 2025-04-24

**Authors:** Brandon Petree, Whitney D. Gannon, Mark Petrovic, John W. Stokes, Enock Adjei, Ryan J. Smith, Caitlin T. Demarest, Konrad Hoetzenecker, Matthew Bacchetta, Anil J. Trindade

**Affiliations:** a Division of Cardiac Surgery, Vanderbilt University Medical Center, Nashville, TN; b Division of Allergy, Pulmonary and Critical Care Medicine, Vanderbilt University Medical Center, Nashville, TN; c Department of Thoracic Surgery, Vanderbilt University Medical Center, Nashville, TN; d Department of Biomedical Engineering, Vanderbilt University, Nashville, TN

**Keywords:** Extracorporeal membrane oxygenation, Bridge-to-transplant, Lung transplantation, Composite allocation score, Waitlist time

## Abstract

The lung composite allocation score (CAS) aims to improve waitlist outcomes for lung transplant candidates by prioritizing characteristics that reflect transplant urgency, including extracorporeal membrane oxygenation bridge to lung transplant (ECMO-BTT). Whether ECMO-BTT has been impacted by CAS is unknown. We analyzed the Organ Procurement and Transplant Network database to examine differences in ECMO-BTT utilization and characteristics and outcomes between transplant recipients who were transplanted one year before and one year after CAS implementation. Lung transplant recipients who received ECMO-BTT in the post-CAS era were younger (*p* < 0.01), more likely to be on ECMO at transplant listing rather than be initiated after (*p* < 0.05), and had shorter waitlist time (*p* < 0.01). Waitlist time was shorter in the post-CAS era among recipients (even non-ECMO) with high allocation scores only. This may have contributed to decreased use of ECMO-BTT in the post-CAS era (*p* = 0.03). One-year post-transplant survival did not differ between eras for ECMO-BTT patients, though was significantly better in the CAS era for non-ECMO-BTT patients (92% vs 90%, *p* < 0.01). We report initial results of ECMO-BTT utilization in the post-CAS era.

## Background

Despite significant growth in lung transplant volume, waitlist mortality is nearly 20%.^1^ The composite allocation score (CAS) was derived to further improve the balance of transplant urgency and post-lung transplant outcomes and has been shown to reduce waitlist time and mortality.^2^, ^3^, ^4^ The change to CAS also aimed to improve the efficiency of donor distribution and equitable access for lung transplant candidates.^2^, ^5^, ^6^ Extracorporeal membrane oxygenation is used to bridge patients with decompensated respiratory failure to lung transplant (ECMO-BTT) to preserve candidacy and optimize outcomes after lung transplant.^7^ Patients who receive ECMO-BTT are those with the greatest urgency for lung transplant due to the severity of critical illness, and the cumulative risk of complications associated with ECMO.^8^ Priority points for ECMO need are now incorporated into CAS.^9^ Therefore, it is possible that implementation of CAS could impact ECMO-BTT utilization, clinical characteristics for whom ECMO-BTT is provided, and early post-transplant outcomes; yet limited data exist. We examined differences in frequency of ECMO-BTT, clinical characteristics, and outcomes among those who received ECMO-BTT before and after CAS implementation. We hypothesized that waitlist time for ECMO-BTT patients in the post-CAS era is lower than during the pre-CAS era.

## Methods

We performed a retrospective registry-based analysis of lung allograft recipients in the Organ Procurement and Transplant Network Standard Transplant Analysis and Research database who were transplanted between March 9, 2022, and March 8, 2024. We excluded multi-organ transplant recipients from the analysis. We stratified the study cohort by transplant recipients who received a lung before and after March 9, 2023 to represent cohorts in the pre-CAS and post-CAS eras. The frequency of ECMO-BTT and variables considered important to medical eligibility for ECMO-BTT were examined. Our primary outcome was waitlist time. We examined waitlist time among the ECMO-BTT cohort and across percentiles of allocation score for the entire cohort. Secondary outcomes included one-year post-transplant survival, receipt of ECMO at 72 hours after transplant, and length of index hospitalization. Between group differences were examined using Chi-square test or Wilcoxon rank-sum test, as appropriate. Kaplan-Meier and multivariable logistic regression was used to assess survival between allocations eras.

## Results

During the study period, 5869 patients received a lung transplant; 81 received multiple organs and were excluded, leaving 5788 recipients for study inclusion. Of the total population, 403 (7.0%) received ECMO-BTT. ECMO-BTT was more frequently utilized in the pre-CAS era [*n* = 212 (7.7%)] than the post-CAS era [*n* = 191 (6.3%) *p* = 0.03]. ECMO-BTT patients were younger in the post-CAS era [41 years (IQR, 54-63)] than the pre-CAS era [48 years (IQR, 38-58) *p* < 0.01]. More patients received ECMO at the time of listing (as opposed to after listing) in the pre-CAS era [*n* = 110 (52%) vs *n* = 80 (42%), *p* < 0.05]. Baseline characteristics among lung transplant recipients were otherwise similar between groups (Table 1). Donor characteristics were similar between groups; however, distances between donors and recipients were significantly greater in the post-CAS era than in the pre-CAS era [539 miles (IQR, 248-797 miles) vs. 179 miles (IQR 93-397 miles), *p* < 0.01].

**Table 1:** Baseline Characteristics for Recipients and Donors and Clinical Outcomes Among the ECMO-BTT Population

	Pre-Composite Allocation Score Era (*n* = 212)	Post-Composite Allocation Score Era (*n* = 191)	*P*
*Recipient Characteristics*			
Age, years	48 (38-58)	41 (54-63)	<0.01
Female sex	90 (42%)	85 (45%)	0.69
White race	158 (75%)	150 (79%)	0.35
ABO blood antigen group			0.93
A	74 (35%)	61 (32%)	
B	23 (11%)	22 (12%)	
O	109 (51%)	103 (54%)	
AB	6 (3%)	5 (3%)	
Body mass index, kg/m^2^	26.7 (21.8-30.0)	25.8 (21.5-29.4)	0.36
Lung disease			0.39
Obstructive	7 (3%)	12 (6%)	
Pulmonary Vascular	13 (6%)	16 (8%)	
Suppurative	6 (3%)	4 (2%)	
Restrictive	186 (88%)	159 (83%)	
Mean pulmonary artery pressure, mmHg	27 (23-39)	27 (22-35)	0.38
Bilateral transplant	202 (95%)	178 (93%)	0.40
Cytomegalovirus mismatch serostatus	44 (21%)	41 (21%)	0.90
Functional status	20 (20-20)	20 (10-20)	0.93
On ECMO prior to listing	110 (52%)	80 (42%)	<0.05
Acute kidney injury on waitlist	16 (8%)	15 (8%)	>0.99
Received dialysis on waitlist	7 (3%)	4 (2%)	0.45
Lung composite allocation score	93.55 (91.78-94.22)	48.21 (45.46-51.08)	N/A
*Donor Characteristics*			
Age, years	35 (25-42)	33 (23-42)	0.28
Extended-criteria donor	19 (9%)	12 (6%)	0.35
Hepatitis C viremic donor	12 (6%)	5 (3%)	0.14
Cigarette use (>20 pack years)	15 (7%)	9 (5%)	0.38
Sex donor/recipient match	149 (70%)	117 (61%)	0.06
ABO match	155 (73%)	129 (68%)	0.23
Predicted total lung capacity donor/recipient	0.97 (0.90-1.10)	1.00 (0.89-1.12)	0.54
Ischemic time, hours	6.3 (5.3-8.0)	6.9 (5.5-8.4)	0.09
Distance, miles	179 (93-397)	539 (248-797)	<0.01
*Outcomes*			
1-year lung transplant recipient survival	175 (83%)	160 (84%)	0.79
Index hospital length of stay, days	36 (22-58)	34 (22-54)	0.44
Wait list time, days	16 (7-37)	9 (4-30)	<0.01
Need for ECMO at 72 hours post-lung transplant	75 (36%)	67 (35%)	>0.99

Abbreviation: ECMO-BTT, extracorporeal membrane oxygenation bridge to transplant.

Data are expressed as frequency with percentage or median with interquartile range unless otherwise specified.

**Figure 1:**
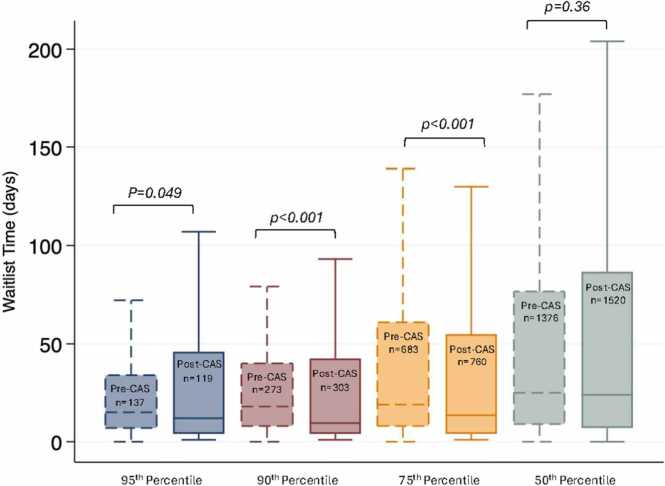
*Waitlist time for lung transplant recipients between pre-CAS and CAS eras, stratified by allocation score percentile.* We determined waitlist time between pre-CAS and CAS eras as a function of illness severity based on allocation score percentile for all lung transplant recipients during the study time period. For patients within the top 95th percentile of illness severity, median waitlist time was 15 days (IQR 7-35) during the pre-CAS era, and 11.5 days (IQR 4-35) during the CAS era, *p* < 0.05. Similarly, for patients within the 90th percentile median waitlist time was 18 days (IQR 8-40) pre-CAS vs 9.5 days (IQR 4-41) after CAS implementation, *p* < 0.01. Median waitlist time was 19 days (IQR 8-61) pre-CAS vs 13.5 days (IQR 4-55) post-CAS, *p* < 0.01, for patients within the top 75th percentile. For patients below the 50th percentile of illness severity, there was no difference in waitlist time between eras [25 days (IQR 9-76) pre-CAS vs 24 days (7-86) post-CAS, *p* = 0.36]. Therefore, CAS implementation is associated with a reduction in waitlist time for patients within the top 75th percentile of illness severity only.

Time on the waitlist for ECMO-BTT recipients was significantly shorter during the post-CAS era than the pre-CAS era [9 days (IQR, 4-30 days) vs 16 days (IQR, 7-37 days), *p* < 0.01]. Waitlist time was significantly shorter post-CAS for lung transplant recipients with allocation scores above the 75th percentile only (Figure 1 and Table 1).

**Figure 2:**
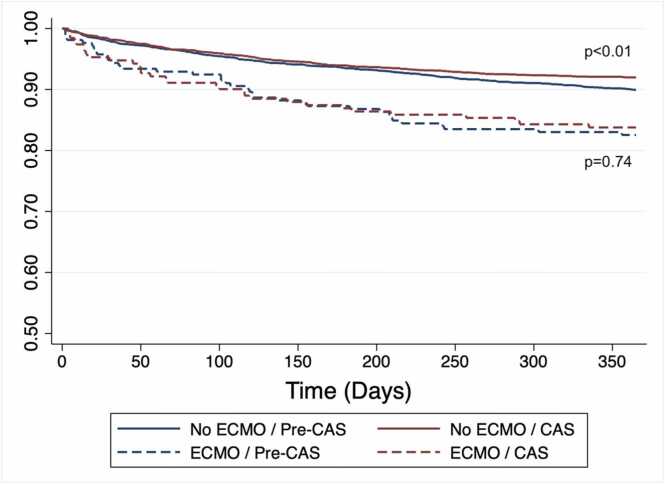
*KM 1-year survival for recipients with and without ECMO-BTT before and after CAS implementation.* In the year preceding the introduction of CAS, there were a total of 2741 lung-only transplant recipients included in the OPTN STAR registry, of whom 212 required ECMO-BTT. One-year survival was 2274/2529 (90%) for those not requiring ECMO-BTT, and 175/212 (83%) for those receiving ECMO-BTT. Post-CAS, 1-year survival was 2626/2856 (92%) for patients without ECMO-BTT and 160/191 (84%) for ECMO-BTT recipients. Between eras there was no significant survival difference for ECMO-BTT (83% vs 84%, *p* = 0.79), though survival increased for patients not requiring ECMO-BTT (90% vs 92%, *p* < 0.01).

Among lung recipients requiring ECMO-BTT, one-year post-transplant survival did not significantly differ between groups (*p* = 0.79), even though one-year survival significantly improved for non-ECMO patients in the CAS era (*p* < 0.01) (Figure 2 and Table 1). This difference remained non-significant after adjusting for recipient age [OR 1.10 (95%CI, 0.65 to 1.87), *p* = 0.71]. There were no differences in index hospital length of stay (*p* = 0.44), need for ECMO at 72 hours post-transplant (*p* > 0.99), or allograft survival (*p* > 0.99) between pre-CAS and post-CAS eras (Table 1).

## Discussion

In this registry study of lung transplant recipients transplanted one year before and after implementation of CAS, ECMO-BTT was provided less frequently and to younger patients in the post-CAS era. Waitlist time among lung transplant recipients who received ECMO-BTT was shorter in the post-CAS era. Waitlist time was also shorter in lung transplant recipients who did not receive ECMO-BTT in the post-CAS era, but only among those with allocation scores above the 75th percentile. Other outcomes were similar between allocation eras for ECMO-BTT recipients, even though one-year survival significantly improved in the CAS era for non-ECMO-BTT recipients.

CAS seeks to reduce time and death on the waitlist and improve access to donor lungs by refining measures that balance urgency with likelihood of favorable post-transplant outcomes.^5^ CAS now considers the potential ramifications of pre-transplant ECMO on wait list urgency and adjusts scores for patients receiving ECMO-BTT.^9^ Therefore, it might be anticipated that the change to CAS would lead to increased provision of ECMO-BTT. On the other hand, a significant reduction in waitlist time in the post-CAS era may avert the need for ECMO-BTT in high-risk candidates, in which case the use of ECMO-BTT would decrease. Our findings show that ECMO is used less often in the post-CAS era. Given the shorter waitlist time post-CAS for sicker patients regardless of ECMO-BTT, we speculate that the prioritization adjustments made for CAS might give way to successfully avoiding ECMO for a greater number of patients than was historically possible. The shorter waitlist time overall may be one reason why one-year survival has improved in the CAS-era for non-ECMO-BTT patients, though this finding may also be explained by improvements in donor management, intra-operative techniques, and post-transplant medical management.

Despite being a well-powered registry analysis, this study has several limitations. The retrospective nature of the study introduces the possibility for selection bias and unmeasured confounding. It would be useful to understand if ECMO duration has changed between eras, though these data are not available in the registry. Moreover, other granular details, including the type of ECMO configuration are also unavailable. We provide an unadjusted risk analysis for the primary outcome; adjusting for risk factors for waitlist time is difficult given the collinearity with the allocation score. This work reports preliminary data and hypothesis generating evidence for future study; no strong conclusions can be drawn from data within the first year of CAS implementation.

We report early results of ECMO-BTT use in the post-CAS era. Future studies to discern how ECMO can be optimally provided to support lung transplant candidates in the post-CAS era are necessary.

## Funding Disclosure

There were no funding sources to support this work.

## Declaration of Competing Interest

The authors declare that they have no known competing financial interests or personal relationships that could have appeared to influence the work reported in this paper.
